# A Rare Case of Creutzfeldt-Jakob Disease in an 80-Year-Old Male

**DOI:** 10.7759/cureus.10038

**Published:** 2020-08-26

**Authors:** Mario Dervishi, Travis Lambert, Maria Markosyan Karapetyan, Nader Warra, Ziyad Iskenderian

**Affiliations:** 1 Internal Medicine, American University of the Caribbean School of Medicine, Cupecoy, SXM; 2 Internal Medicine, Ascension Providence Hospital, Southfield, USA; 3 Neurology, Ascension Providence Hospital, Southfield, USA

**Keywords:** creutzfeldt-jakob disease, prion diseases, neurodegenerative disorders

## Abstract

Creutzfeldt-Jakob disease (CJD) is a rare, rapid and fatal human prion disease that causes neurodegeneration. Rapidly progressive dementia, quick involuntary muscle jerking and specific radiographic and laboratory findings are characteristic of the disease. CJD should not be ruled even if the clinical presentation is outside the common age range. Herein we present a case of an 80-year-old man with probable diagnosis of CJD. The absolute diagnosis of CJD can only be confirmed post-mortem with a brain biopsy.

## Introduction

Prion diseases are a cluster of neurodegenerative pathologies caused by misfolding of proteins called prion [1]. Creutzfeldt-Jakob disease (CJD) is the most common form and accounts for more than 90% of human prion diseases, although it is still rare with 350 cases per year reported in the United States [2]. This disease typically presents with rapid course of symptomatology and the unfortunate, inevitable fate is death. Amongst subtypes (sporadic, familial, iatrogenic and variant), sporadic CJD is the most common form seen in 85%-90% of cases. Disease most commonly affects people of ages 50-70 years, with both genders equally affected [3].

The main identifying clinical features of CJD are rapidly progressive dementia and multifocal neurological findings such as myoclonus, visual disturbances, cerebellar, and pyramidal/extrapyramidal signs. The disease rapidly progresses to cognitive and functional impairment, akinetic mutism in the late stage, and eventually death, most often within a year or less from the disease onset [3-6]. Along with clinical presentation, electroencephalography (EEG), magnetic resonance imaging (MRI), and cerebrospinal fluid (CSF) assays, are necessary in order to rule out other neurodegenerative disorders. The differential diagnosis includes non-prion neurodegenerative diseases like Alzheimer's dementia, Lewy body dementia, and frontotemporal dementia. Most common vascular and metabolic etiologies of dementia must be ruled out first.

## Case presentation

An 80-year-old male with a past medical history of a prior stroke with associated left-sided weakness, and multiple surgical procedures presented to the neurologist outpatient clinic with significant neurological deterioration and 20-pound weight loss over 3-4 months. As per patient’s family, he abruptly developed sudden onset of confusion, memory loss, aphasia, and dementia-type behavior.

The neurological exam revealed an awake patient, unable to follow directions. The patient showed signs of akinetic mutism; nonverbal and unable to mimic or model commands or gestures. Cranial nerves were grossly intact, and he was able to move all four extremities against gravity. The patient presented with upper extremity rigidity. During the evaluation, increased tone in all four limbs and myoclonus were present. Reflexes were brisk and symmetric. The patient was ataxic and exhibited gait and coordination dysfunction. Pain reflex was intact in all four extremities.

**Figure 1:**
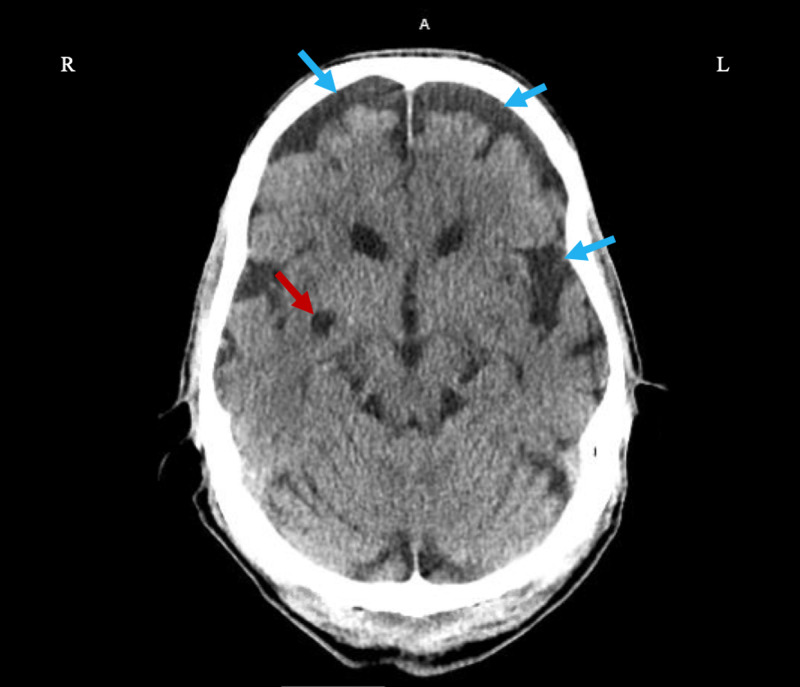
Axial Non-Contrast Head CT Diffuse brain atrophy (blue arrows) and old left lacunar infarct (red arrow).

A non-contrast CT of the brain was performed to rule out ischemic or hemorrhagic etiologies. An acute intracranial hemorrhage or major vascular territorial infarcts were absent. Diffuse atrophy, and an old, right lacunar infarct were appreciated (Figure 1).

**Figure 2:**
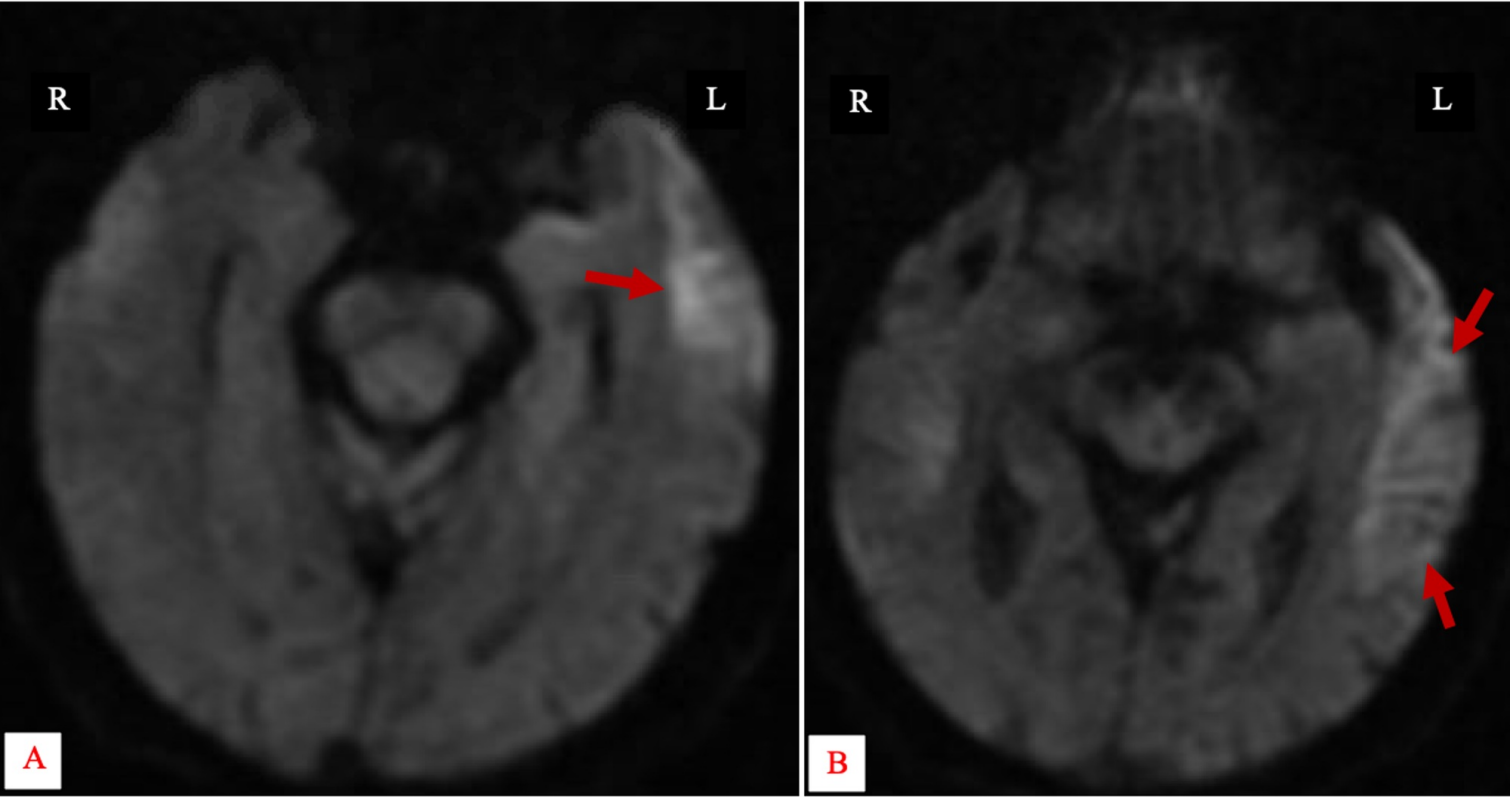
Axial Non-Contrast Diffusion Weighted MR Image of the Brain Restricted diffusion involving the left temporal lobe (red arrows).

The brain MRI was limited by patient’s inability to tolerate the test and the study was markedly degraded by motion artifact. Limited results were obtained only from axial diffusion weighted images showing changes in the left temporal lobe, suspicious of a sub-acute infarct (Figure 2A, 2B).

Given the patient’s clinical manifestations of myoclonus, mutism, and acute neurological deterioration, a prion disease was suspected, and further evaluation with EEG and lumbar puncture was indicated. EEG was performed at an outpatient clinic and dictated findings were nonspecific in etiology and could be consistent with an underlying encephalopathic state such as that caused by toxic, metabolic or vascular abnormalities.

**Figure 3:**
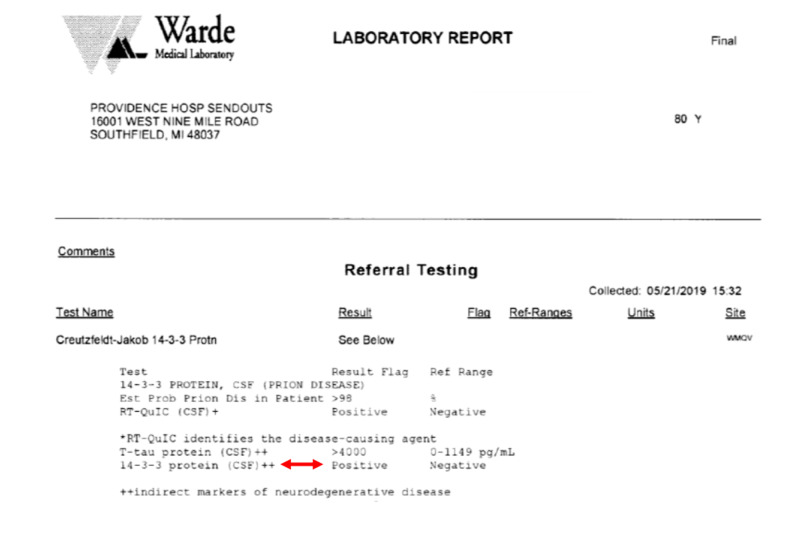
Laboratory Report Positive cerebrospinal fluid (CSF) analysis for 14-3-3 protein (doubleheader red arrow).

A lumbar puncture was performed, and in hospital CSF analysis showed normal protein and glucose levels with no organisms present and within normal limits white blood cells, ruling out infectious etiologies. Herpes simplex 1 and 2 and venereal disease research laboratory (VDRL), were both negative. A sample of patient’s CSF was sent to an offsite laboratory, tested for 14-3-3 protein and the results came back positive (Figure 3).

Unfortunately, the patient deteriorated quickly due to disease progression and shortly thereafter expired. A brain autopsy was recommended to definitely confirm the diagnosis, however the family declined.

## Discussion

Clinical presentation of CJD overlaps with other infectious and metabolic causes of dementia. It also overlaps with other neurodegenerative disorders. A careful differential diagnosis is imperative to rule out treatable causes and avoid unnecessary tests. The most common neurodegenerative disorders that present with dementia are Alzheimer's disease (AD), dementia with Lewy body (DLB) and frontotemporal dementia (FTD) [3,4]. In contrast to CJD, the latter etiologies of dementia typically are insidious in onset, and progress very slowly for many years.

Creutzfeldt-Jakob disease is the rarest amongst the neurodegenerative diseases, and usually presents in ages 50-70 years, with both genders equally affected [3,7]. Rare presentation outside the common age-range is possible as seen in our case. Hence, it is important to not rule out CJD if patient presentation is of clinical significance. Abrupt onset of confusion, depression, apathy, memory loss, aphasia and progressive dementia are non-specific findings seen in CJD. Myoclonus, in particular when provoked by startle, is specific to CJD [1,3,5]. In addition, nystagmus, ataxia, hypokinesia, bradykinesia, dystonia, rigidity or akinetic mutism is present in most patients. Psychotic features, like visual hallucinations, can also be present in some patients but were absent in this case. Specific findings of high signal in caudate, putamen can be seen on brain MRI, which was limited in this case due to patient’s inability to tolerate the test. Periodic sharp wave complexes on EEG are commonly seen in CJD. 14-3-3 protein in CSF analysis is a specific finding of CJD supporting the diagnostic criteria.

Overall, our case presentation fits the CDC diagnostic criteria for probable diagnosis of sporadic CJD by clinical presentation and positive CSF analysis for 14-3-3 protein. Review of literature reports very few case presentations of CJD outside the age groups reported above. Uncommon age presentation in this 80-year-old patient recognizes the possibility of CJD outside the commonly affected age groups.

Unfortunately, there is no current treatment of CJD. However, early diagnosis of the disease allows patients and their families’ time to understand the disease course and arrange for advanced directives. Early diagnosis is also important to be sure the prion does not spread to family, health professionals and mortuary staff.

## Conclusions

Rare presentation of CJD can be seen in elderly patients outside the common age range. If the clinical criteria are met and most common etiologies of dementia are ruled out first, clinicians must be aware of this uncommon presentation to make proper diagnosis and avoid unnecessary tests.
